# An NF90/long noncoding RNA-LET/miR-548k feedback amplification loop controls esophageal squamous cell carcinoma progression

**DOI:** 10.7150/jca.30816

**Published:** 2019-08-28

**Authors:** Jianqing Lin, Zhiyao Chen, Shanhu Wu, Wenbo Huang, Feng Chen, Zhijun Huang

**Affiliations:** Department of Surgical Oncology, the Second Affiliated Hospital of Fujian Medical University, Quanzhou 362000, Fujian, China; Jianqing Lin and Zhiyao Chen are co-first authors.

**Keywords:** esophageal squamous cell carcinoma, NF90, miR-548k, long noncoding RNA, feedback loop

## Abstract

In our previous study we have found that miR-548k has oncogenic roles in esophageal squamous cell carcinoma (ESCC) via repressing long noncoding RNA (lncRNA)-LET and further upregulating nuclear factor 90 (NF90). However, the upstream factors controlling miR-548k expression are still unknown. In this study, we found NF90 directly binds pri-miR-548k, increases the stability of pri-miR-548k, and upregulates the expression of pri-miR-548k and miR-548k. Therefore, NF90, miR-548k and lncRNA-LET forms a feedback loop. Gain-of-function and loss-of-function assays demonstrated that in accordance with the roles of miR-548k, NF90 also promotes ESCC cell proliferation and migration. Furthermore, we verified the regulatory feedback loop between NF90, miR-548k, and lncRNA-LET. We found NF90 upregulated miR-548k and downregulated lncRNA-LET. miR-548k downregulated lncRNA-LET and upregulated NF90. lncRNA-LET downregulated NF90 and miR-548k. Through the reciprocal regulations between each other, the NF90/miR-548k/lncRNA-LET feedback loop controls the expressions of NF90 targets (HIF-1α and VEGF), miR-548k targets (KLF10 and EGFR), and lncRNA-LET target (p53). Further functional assays demonstrated that activation of the NF90/miR-548k/lncRNA-LET feedback loop via simultaneously overexpressing NF90 and miR-548k and simultaneously depleting lncRNA-LET significantly promotes ESCC cell proliferation and migration *in vitro* and ESCC tumor growth *in vivo*. Targeting the NF90/miR-548k/lncRNA-LET feedback loop via simultaneously depleting NF90 and miR-548k and simultaneously overexpressing lncRNA-LET significantly inhibits ESCC cell proliferation and migration *in vitro* and ESCC tumor growth *in vivo*. In summary, our findings identified a crucial oncogenic NF90/lncRNA-LET/miR-548k feedback amplification loop, which may be promising therapeutic targets for ESCC.

## Introduction

Esophageal cancer is one of the most common cancer and the sixth leading cause of cancer-related death worldwide with an estimated 572.0 thousand new cases and 508.6 thousand deaths in 2018 1, 2. Esophageal squamous cell carcinoma (ESCC) is the predominant histopathological subtype of esophageal cancer 3, 4. Although advances have been made in multiple therapeutic strategies, the overall 5-year survival rate of ESCC is still very poor, with about 10% to 25% 5. The poor prognosis of ESCC is associated with later diagnosis of ESCC patients which are not suitable for surgical resection 6. Except surgical resection, other therapeutic strategies are relative less efficient due to the lack of the understanding of underlying molecular mechanisms mediating the initiation and progression of ESCC 7.

Genome sequencing, exome sequencing, transcriptome sequencing, and array comparative genomic hybridization of ESCC tissues have identified many mutated genes, genomic amplified or deleted regions, and aberrantly expressed transcripts 7, 8. Among these aberrantly expressed transcripts, not only mRNAs, but also many non-coding RNAs were identified 4, 9. MicroRNA (miRNA) is a class of small non-coding RNA with about 21-25 nucleotides in length 10, 11. Accumulating evidences have demonstrated that miRNAs are frequently dysregulated in many diseases, particular in cancers, and have crucial roles in various pathophysiological processes 12-17. miRNAs induce their targets transcripts degradation and/or translation repression via base pairing with target transcripts 18-20. In our previous study, we have found miR-548k as an oncogenic and upregulated miRNA in ESCC 21. However, the factors regulating miR-548k expression in ESCC are still not fully understood.

Long noncoding RNA (lncRNA) is another class of non-coding RNA with more than 200 nucleotides in length and no protein coding potential 22-24. Recent advances in genome and transcriptome sequencing have found more than 58,000 lncRNAs in human cells 25. The number of genes coding proteins is only about 21,000 25. Among these lncRNAs, many are revealed to be dysregulated in cancers and implicated in almost every aspect of cancers, including cell proliferation, cell cycle, apoptosis, migration, invasion, metastasis, angiogenesis, drug-resistance, and so on 26-35. In accordance with miRNAs, lncRNAs are also considered as important regulatory RNAs in ESCC 36, 37. In our previous study, we have identified lncRNA-LET as a tumor suppressor in ESCC 21. Furthermore, we found lncRNA-LET as a direct target of miR-548k 21. Nuclear factor 90 (NF90) was identified as a downstream target of lncRNA-LET 21.

NF90 is an RNA binding protein, which has been reported to regulate the stability and translation of bound RNAs and miRNA biogenesis 38-40. Intriguingly, in this study, we further found NF90 also bound pri-miR-548k and upregulated the expression of pri-miR-548k and miR-548k. Thus, NF90, miR-548k, lncRNA-LET forms a positive feedback loop in ESCC. We further investigated the biological roles and mechanisms of action of the NF90/miR-548k/lncRNA-LET regulatory loop in ESCC.

## Materials and Methods

### Cell culture and treatment

ESCC cell lines KYSE30 and Eca-109 were acquired from the Institute of Biochemistry and Cell Biology of the Chinese Academy of Sciences (Shanghai, China). KYSE30 and Eca-109 cells were cultured in RPMI 1640 medium (Invitrogen, Carlsbad, CA, USA) supplemented with 10% fetal bovine serum (Invitrogen) in a humidified incubator containing 5% CO_2_ at 37°C. Where indicated, the ESCC cells were treated with 50 μM α-amanitin (Sigma-Aldrich, Saint Louis, MO, USA) for the indicated time to inhibit RNA polymerase II-mediated transcription.

### Plasmids construction and transfection

The complementary DNA (cDNA) coding NF90 was PCR-amplified with the *PfuUltra* II Fusion HS DNA Polymerase (Agilent Technologies, Santa Clara, CA, USA) and the primers 5'-GGGGTACCGTTGAAGTATTGATAACACCAA-3' (sense) and 5'-GGGTTTAAACACAAACCATTGAAGACACGG-3' (anti-sense). Next, the PCR products were subcloned into the *Kpn* I and *Pme* I sites of the pcDNA3.1 plasmid (Invitrogen), named as pcDNA3.1-NF90. lncRNA-LET overexpression plasmid pcDNA3.1-LET was constructed in our previous study 21. To inhibit NF90 expression, two independent cDNA oligonucleotides specifically targeting NF90 were designed as previously described 41, synthesized by GenePharma (Shanghai, China), and inserted into the supersilencing^TM^ shRNA expression vector pGPU6/Hygro (GenePharma), named as shRNA-NF90-1 and shRNA-NF90-2. The shRNAs target sites were 5'-GCUCAAAGCUGUGUCCGACUGGA-3' (shRNA-NF90-1) and 5'-AAGCCACUGAUGCUAUUGGGC-3' (shRNA-NF90-2). To inhibit lncRNA-LET expression, two independent cDNA oligonucleotides specifically targeting lncRNA-LET were designed as we previously described 21, synthesized by GenePharma (Shanghai, China), and inserted into the supersilencing^TM^ shRNA expression vector pGPU6/Hygro (GenePharma), named as shRNA-LET-1 and shRNA-LET-2. The shRNAs target sites were 5'-TGGGAGTAAAGGGAAAGAGTT-3' (shRNA-LET-1) and 5'-GTGCATGTGGTAGGTTAGATT-3' (shRNA-LET-2). A scrambled shRNA was used as negative control, named as shRNA-NC. The transfection of pcDNA3.1, pcDNA3.1-NF90, pcDNA3.1- LET, shRNA-NC, shRNA-NF90-1, shRNA-NF90-2, shRNA-LET-1, and shRNA-NF90-1 were undertaken with Lipofectamine 3000 (Invitrogen) according to the protocol.

### Lentivirus production and stable cell lines construction

Recombinant hsa-miR-548k overexpression lentiviruses, recombinant has-miR-548k inhibition lentiviruses, and their respective negative control lentiviruses were purchased from GenePharma. For construction of NF90 overexpressed KYSE30 cells, KYSE30 cells were transfected with pcDNA3.1-NF90 and selected with neomycin for four weeks. For construction of NF90 depleted Eca-109 cells, Eca-109 cells were transfected with shRNA-NF90-1 or shRNA-NF90-2, and selected with hygromycin for four weeks. For construction of lncRNA-LET overexpressed Eca-109 cells, Eca-109 cells were transfected with pcDNA3.1-LET and selected with neomycin for four weeks. For construction of lncRNA-LET depleted KYSE30 cells, KYSE30 cells were transfected with shRNA-LET-1 or shRNA-LET-2, and selected with hygromycin for four weeks. For construction of miR-548k overexpressed KYSE30 cells, KYSE30 cells were infected with 2×10^6^ transducing units of miR-548k overexpression lentiviruses and selected with puromycin for four weeks. For construction of miR-548k depleted Eca-109 cells, Eca-109 cells were infected with miR-548k inhibition lentiviruses and selected with puromycin for four weeks. For construction of NF90 overexpressed and miR-548k simultaneously depleted KYSE30 cells, NF90 overexpressed KYSE30 cells were infected with miR-548k inhibition lentiviruses and selected with neomycin and puromycin for four weeks. For construction of NF90 and miR-548k simultaneously overexpressed and lncRNA-LET simultaneously depleted KYSE30 cells, NF90 overexpressed KYSE30 cells were transfected with shRNA-LET-2 and selected with neomycin and hygromycin for four weeks. Then, the cells were further infected with 2×10^6^ transducing units of miR-548k overexpression lentiviruses and selected with puromycin, neomycin and hygromycin for four weeks. For construction of NF90 and miR-548k simultaneously depleted and lncRNA-LET simultaneously overexpressed Eca-109 cells, NF90 depleted Eca-109 cells were transfected with pcDNA3.1-LET and selected with neomycin and hygromycin for four weeks. Then, the cells were further infected with 2×10^6^ transducing units of miR-548k inhibition lentiviruses and selected with puromycin, neomycin and hygromycin for four weeks.

### Western blot

Total proteins were extracted from indicated ESCC cells with RIPA buffer (Beyotime, Jiangsu, China) added with protease inhibitors (Beyotime). Protein concentrations were determined by BCA assay using the BCA Protein Assay Kit (Beyotime). Equal amounts of proteins were separated by 10% sodium dodecyl sulfate-polyacrylamide gel electrophoresis, followed by being transferred to nitrocellulose membrane (Millipore, Bedford, MA, USA). After being blocked, the membranes were incubated with NF90 specific primary antibody (ab131004, Abcam, Hong Kong, China) and β-actin specific primary antibody (66009-1-Ig, Proteintech, Rosemont, IL, USA). After three washes, the membranes were incubated with IRDye 700CW goat anti-mouse IgG (Li-Cor, Lincoln, NE, USA) and IRDye 800CW goat anti-rabbit IgG (Li-Cor), followed by being scanned on an Odyssey infrared scanner (Li-Cor).

### RNA extraction, reverse transcription, and quantitative real time polymerase chain reaction (qRT-PCR)

Total RNA was extracted from indicated ESCC cells with the TRIzol Regent (Invitrogen) following the protocol. The quality of RNA was assessed by formaldehyde agarose gel electrophoresis and quantified by NanoDrop UV-Vis Spectrophotometer. After being treated with DNase I (Takara, Dalian, China) to remove genomic DNA, the purified RNA was subjected to reverse transcription with the M-MLV Reverse Transcriptase (Invitrogen) following the protocol. qRT-PCR was undertaken on ABI StepOnePlus Real-Time PCR System (Applied Biosystems, Foster City, CA, USA). The expressions of mRNAs and lncRNAs were measured using SYBR^®^ Premix Ex Taq™ II (Takara) with the standard SYBR Green protocol. The expressions of miR-548k, pri-miR-548k, and pri-miR-21 were measured using TaqMan^TM^ MicroRNA Assay (Applied Biosystems) and TaqMan^TM^ Pri-miRNA Assay (Applied Biosystems) following the manufacturer's protocols. The sequences of the primers were as follows: for lncRNA-LET, 5'-TGAGATGCTGGAATGATG-3' (sense) and 5'-GGCTAAAGAAGGAAAAGG-3' (antisense); for HIF-1α, 5'-ATGAAGTGTACCCTAACTAGCCG-3' (sense) and 5'-CCAAGCAGGTCATAGGTGGTTTC-3' (antisense) 42; for VEGF, 5'-CGCAAGAAATCCCGGTATAA-3' (sense) and 5'-AAATGCTTTCTCCGCTCTGA-3' (antisense) 39; for KLF10, 5'-GGAGGAAAGAATGGAAATG-3' (sense) and 5'-GTCAGAAGGACTGTAAGG-3' (antisense); for EGFR, 5'-TTCACACATACTCCTCCTC-3' (sense) and 5'-TCTCCATCACTTATCTCCT-3' (antisense); for p53, 5'-GTGTGGTGGTGCCCTATGA-3' (sense) and 5'-GTGAGGCTCCCCTTTCTTG-3' (antisense); for 18S rRNA, 5'-ACACGGACAGGATTGACAGA-3' (sense) and 5'-GGACATCTAAGGGCATCACA-3' (antisense); for GAPDH, 5'-GGAGCGAGATCCCTCCAAAAT-3' (sense) and 5'-GGCTGTTGTCATACTTCTCATGG-3' (antisense). GAPDH was employed as endogenous control for the quantification of mRNAs, lncRNAs, and pri-miRNAs. U6 was employed as endogenous control for the quantification of miRNAs. The PCR efficiency was determined using qRT-PCR with standard curve method. The quantifications of RNAs were calculated using the comparative Ct method.

### RNA Immunoprecipitation (RIP) assay

RNA Immunoprecipitation (RIP) assay was undertaken in Eca-109 cells with the EZ-Magna RIP™ RNA Binding Protein Immunoprecipitation Kit (Millipore) and NF90 specific primary antibody (ab131004, Abcam) following the protocol. The retrieved RNA was quantified using qRT-PCR as above described.

### Cell proliferation assay

Cell proliferation was evaluated using Glo cell viability assay and Ethynyl deoxyuridine (EdU) incorporation assay as we previously described 21. Briefly, for Glo cell viability assay, indicated ESCC cells were plated per well into 96-well plates and maintained for the indicated time. At indicated time points, the luminescence values were acquired with the CellTiter-Glo^®^ Luminescent Cell Viability Assay (Promega, Madison, WI, USA) following the protocol. Then the luminescence values were used to plot the cell proliferation curves. EdU incorporation assay was undertaken using the EdU kit (Roche, Mannheim, Germany) following the protocol. The results were collected and quantified by the Zeiss fluorescence photomicroscope (Carl Zeiss, Oberkochen, Germany).

### Cell migration assay

Cell migration was evaluated by transwell assay as we previously described 21. Briefly, indicated ESCC cells re-suspended in serum-free medium with 1 μg/mL Mitomycin C to inhibit cell proliferation were seeded in the upper chamber of Millicell transwell chamber (8 μm pore size, Millipore). The lower chamber was supplemented with complete medium. After incubation for 48 hours, the cells on the upper chamber were scraped off, and the migratory cells on the bottom of the chamber were fixed, stained, and quantified using the Zeiss photomicroscope (Carl Zeiss).

### Xenograft model

To construct ESCC xenograft model, 2×10^6^ indicted ESCC cells were subcutaneously injected into 5-weeks-old male athymic BALB/c nude mice (SLRC Laboratory Animal Center, Shanghai, China). Subcutaneous tumor volumes were detected every four or seven days using a caliper and calculated by the formula V = 0.5 × L × W^2^ (L, length; W, width). At indicated time, the nude mice were sacrificed and subcutaneous xenografts were resected and weighted. The use of animals was reviewed and approved by the Review Board of the Second Affiliated Hospital of Fujian Medical University.

### Statistical analysis

Statistical analyses were undertaken with the GraphPad Prism Software. Comparisons between groups were subjected to Student's *t*-test, one-way ANOVA followed by Dunnett's multiple comparison test, or Mann-Whitney *U* test as indicated. *P* < 0.05 was considered as statistically significant.

## Results

### NF90 upregulates miR-548k expression

**Fig 1:**
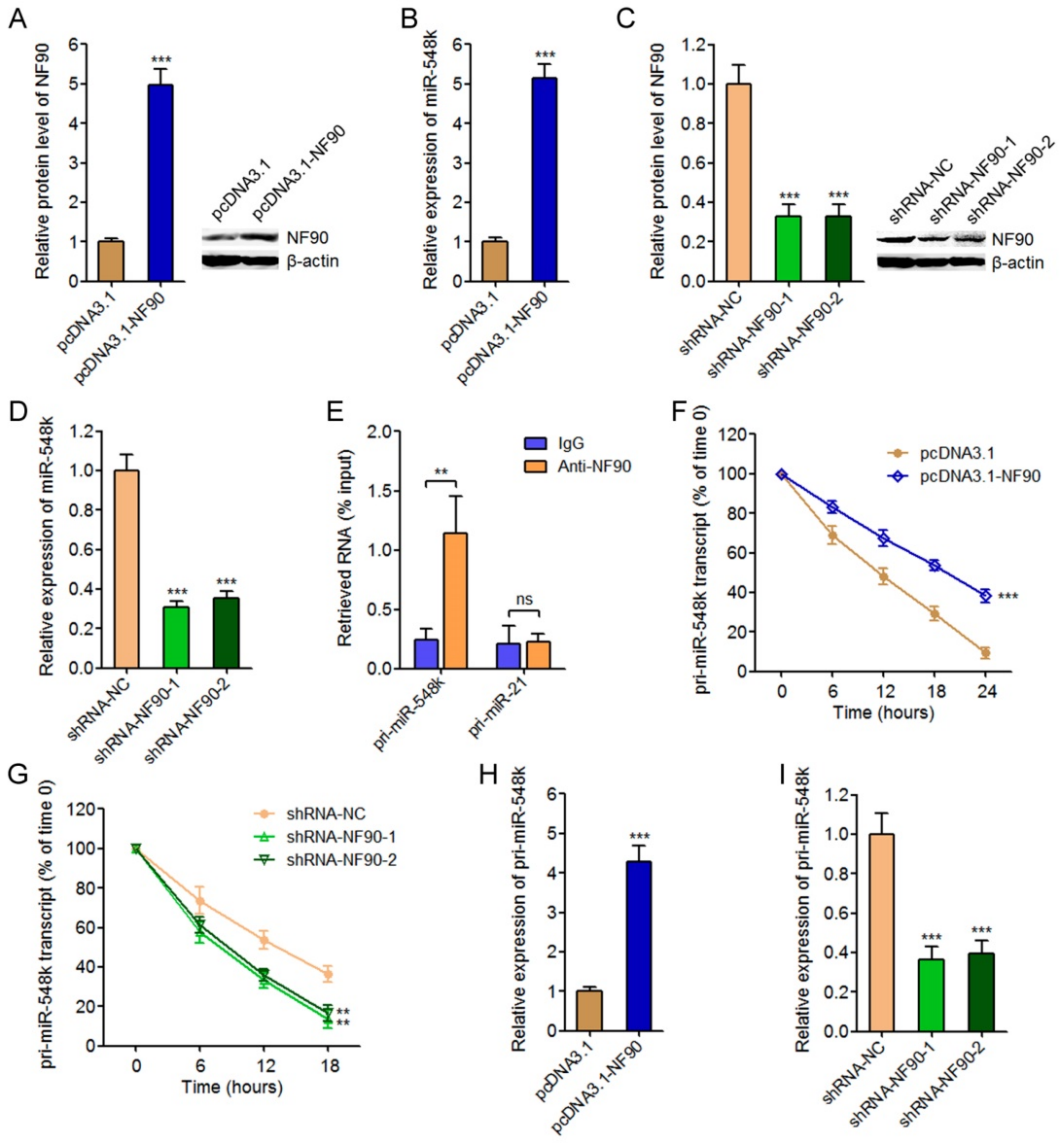
** The regulatory roles of NF90 on miR**-**548k.** (A) After transfection of NF90 overexpression or control plasmids into KYSE30 cells, NF90 expression were measured by western blot. (B) After transfection of NF90 overexpression or control plasmids into KYSE30 cells, miR-548k expression were measured by qRT-PCR. (C) After transfection of NF90 specific or control shRNAs into Eca-109 cells, NF90 expression were measured by western blot. (D) After transfection of NF90 specific or control shRNAs into Eca-109 cells, miR-548k expression were measured by qRT-PCR. (E) RIP assay followed by qRT-PCR was performed to detect the specific enrichment of pri-miR-548k with NF90 specific antibody compared with nonspecific IgG. pri-miR-21 was used as negative control. (F) After transfection of NF90 overexpression or control plasmids into KYSE30 cells, the stability of pri-miR-548k transcript over time was evaluated by qRT-PCR relative to time 0 after blocking new RNA synthesis with α-amanitin and normalized to 18S rRNA (transcribed by RNA polymerase I and not influenced by α-amanitin). (G) After transfection of NF90 specific or control shRNAs into Eca-109 cells, the stability of pri-miR-548k transcript over time was evaluated by qRT-PCR relative to time 0 after blocking new RNA synthesis with α-amanitin and normalized to 18S rRNA. (H) After transfection of NF90 overexpression or control plasmids into KYSE30 cells, pri-miR-548k expression was measured by qRT-PCR. (I) After transfection of NF90 specific or control shRNAs into Eca-109 cells, pri-miR-548k expression was measured by qRT-PCR. Results are presented as mean ± S.D. (n = 3). **P < 0.01, ***P < 0.001, ns, not significant, by Student's *t*-test (A, B, E, F and H) or one-way ANOVA followed by Dunnett's multiple comparison test (C, D, G and I).

To investigate the effects of NF90 on miR-548k, we overexpressed NF90 in KYSE30 cells via transfecting NF90 overexpression plasmids (**Figure 1A**). The expression of miR-548k in these transfected cells was measured by qRT-PCR. As displayed in **Figure 1B**, enhanced expression of NF90 significantly upregulated miR-548k expression. Furthermore, we inhibited NF90 in Eca-109 cells via transfecting NF90 specific shRNAs, and the results displayed that depletion of NF90 significantly downregulated miR-548k expression (**Figure 1C** and** 1D**). As an RNA binding protein, NF90 has been reported to bind mRNAs and/or pri-miRNAs and change their stabilities 38, 40. Thus, we next investigated whether NF90 also regulates pri-miR-548k. RIP assays using NF90 specific antibody displayed that NF90 specifically interacted with pri-miR-548k, but not pri-miR-21 (**Figure 1E**). Next, we investigated the effects of NF90 on pri-miR-548k stability. 48 hours after transient transfection of NF90 overexpression or control plasmids into KYSE30 cells, the transfected cells were treated with 50 μM α-amanitin to block new RNA synthesis. The loss of pri-miR-548k transcript overtime was measured using qRT-PCR. As displayed in **Figure 1F**, enhanced expression of NF90 significantly elongated the half-life of pri-miR-548k transcript. Similarly, 48 hours after transient transfection of NF90 specific shRNAs or control shRNAs into Eca-109 cells, the transfected cells were treated with 50 μM α-amanitin and the loss of pri-miR-548k transcript overtime was measured. The results displayed that depletion of NF90 significantly shortened the half-life of pri-miR-548k transcript (**Figure 1G**). In addition, the effects of NF90 on pri-miR-548k expression were investigated. As displayed in **Figure 1H** and **1I**, enhanced expression of NF90 upregulated pri-miR-548k expression, and while depletion of NF90 downregulated pri-miR-548k expression. Collectively, these data suggested that NF90 interacted with pri-miR-548k, increased pri-miR-548k stability, and upregulated the expression of pri-miR-548k and miR-548k.

### NF90 promotes ESCC cell proliferation and migration

**Fig 2:**
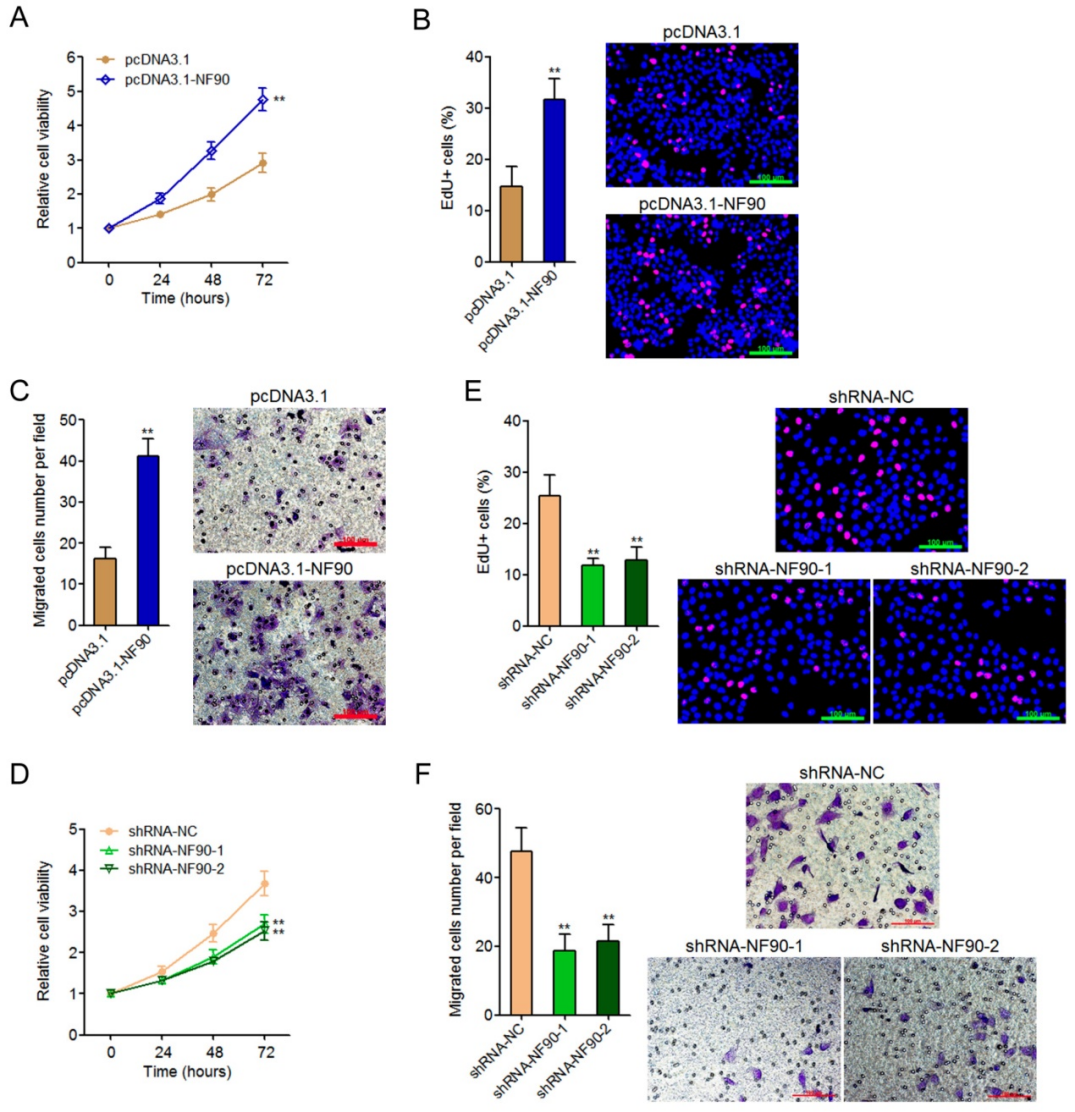
** The biological roles of NF90 in ESCC cell proliferation and migration.** (A) Glo cell viability assay was performed to detect cell viability of NF90 overexpressed and control KYSE30 cells. (B) EdU incorporation assay was performed to detect cell proliferation of NF90 overexpressed and control KYSE30 cells. The red color represents EdU-positive and proliferation active cells. Scale bars, 100 μm. (C) Transwell assay was performed to detect cell migration of NF90 overexpressed and control KYSE30 cells. Scale bars, 100 μm. (D) Glo cell viability assay was performed to detect cell viability of NF90 depleted and control Eca-109 cells. (E) EdU incorporation assay was performed to detect cell proliferation of NF90 depleted and control Eca-109 cells. The red color represents EdU-positive and proliferation active cells. Scale bars, 100 μm. (F) Transwell assay was performed to detect cell migration of NF90 depleted and control Eca-109 cells. Scale bars, 100 μm. Results are presented as mean ± S.D. (n = 3). **P < 0.01 by Student's *t*-test (A-C) or one-way ANOVA followed by Dunnett's multiple comparison test (D-F).

To explore the biological roles of NF90 in ESCC, we constructed NF90 overexpressed KYSE30 cells via transfecting NF90 overexpression plasmids. Glo cell viability assay displayed that enhanced expression of NF90 increased cell viability of KYSE30 cells (**Figure 2A**). EdU incorporation assay further displayed that enhanced expression of NF90 promoted cell proliferation of KYSE30 cells (**Figure 2B**). Transwell migration assay displayed that enhanced expression of NF90 promoted cell migration of KYSE30 cells (**Figure 2C**). In addition, we also constructed NF90 depleted Eca-109 cells via transfecting NF90 specific shRNAs. Glo cell viability assay displayed that depletion of NF90 decreased cell viability of Eca-109 cells (**Figure 2D**). EdU incorporation assay further displayed that depletion of NF90 inhibited cell proliferation of Eca-109 cells (**Figure 2E**). Transwell migration assay displayed that depletion of NF90 inhibited cell migration of Eca-109 cells (**Figure 2F**). Collectively, these data demonstrated that NF90 promotes ESCC cell proliferation and migration.

### Inhibition of miR-548k abolishes the oncogenic roles of NF90 in ESCC

**Fig 3:**
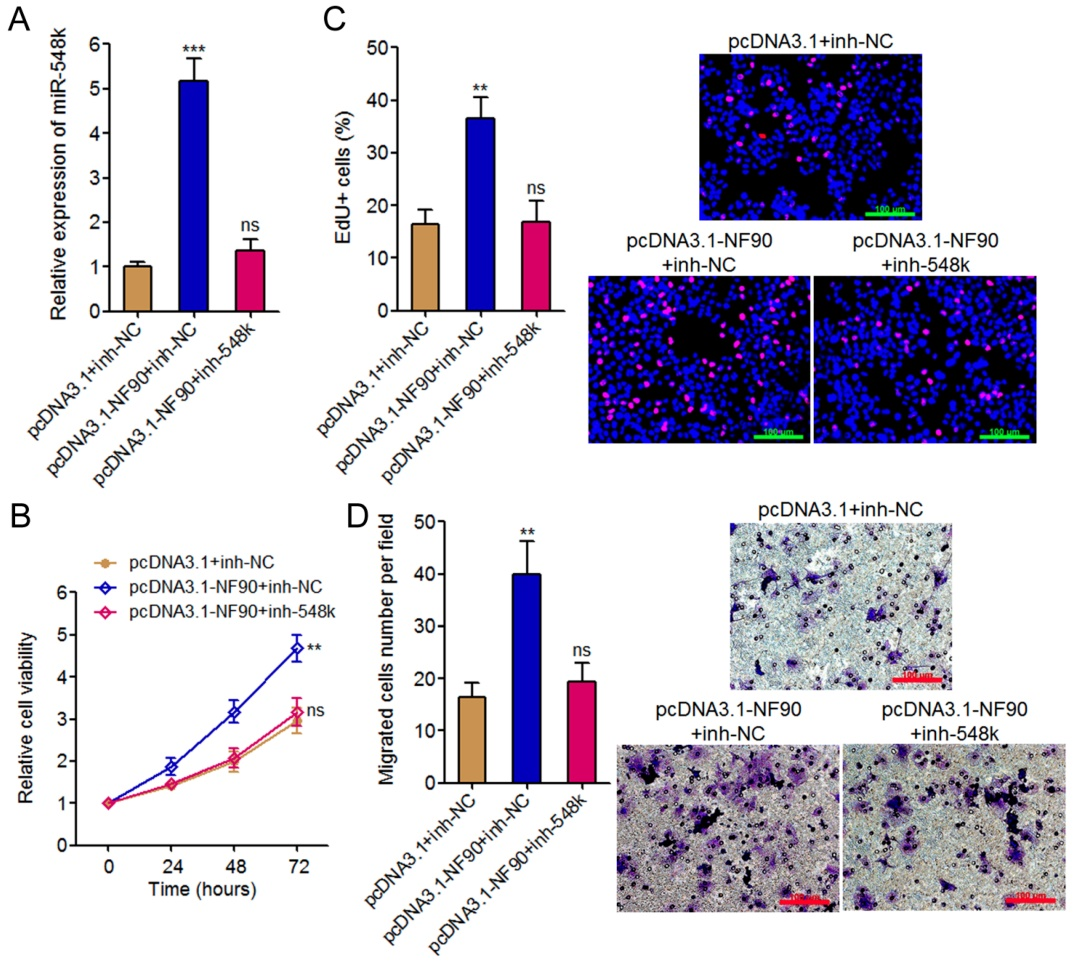
** The roles of NF90 in ESCC cell proliferation and migration are miR**-**548k dependent.** (A) miR-548k expression in NF90 overexpressed and miR-548k simultaneously depleted and control KYSE30 cells was measured by qRT-PCR. (B) Glo cell viability assay was performed to detect cell viability of NF90 overexpressed and miR-548k simultaneously depleted and control KYSE30 cells. (C) EdU incorporation assay was performed to detect cell proliferation of NF90 overexpressed and miR-548k simultaneously depleted and control KYSE30 cells. The red color represents EdU-positive and proliferation active cells. Scale bars, 100 μm. (D) Transwell assay was performed to detect cell migration of NF90 overexpressed and miR-548k simultaneously depleted and control KYSE30 cells. Scale bars, 100 μm. Results are presented as mean ± S.D. (n = 3). **P < 0.01, ***P < 0.001, ns, not significant, by one-way ANOVA followed by Dunnett's multiple comparison test.

To investigate whether the oncogenic roles of NF90 in ESCC were dependent on the positive regulation of miR-548k, we inhibited miR-548k expression in NF90 overexpressed KYSE30 cells via infecting miR-548k inhibition lentiviruses (**Figure 3A**). Glo cell viability assay displayed that inhibition of miR-548k abolished the increasing of cell viability caused by enhanced expression of NF90 (**Figure 3B**). EdU incorporation assay displayed that inhibition of miR-548k abolished the pro-proliferative roles of NF90 (**Figure 3C**). Transwell migration assay displayed that inhibition of miR-548k abolished the pro-migratory roles of NF90 (**Figure 3D**). Collectively, these data demonstrated that the positive regulation of miR-548k at least partially mediates the oncogenic roles of NF90 in ESCC.

### The feedback amplification loop between NF90, miR-548k, and lncRNA-LET

**Fig 4:**
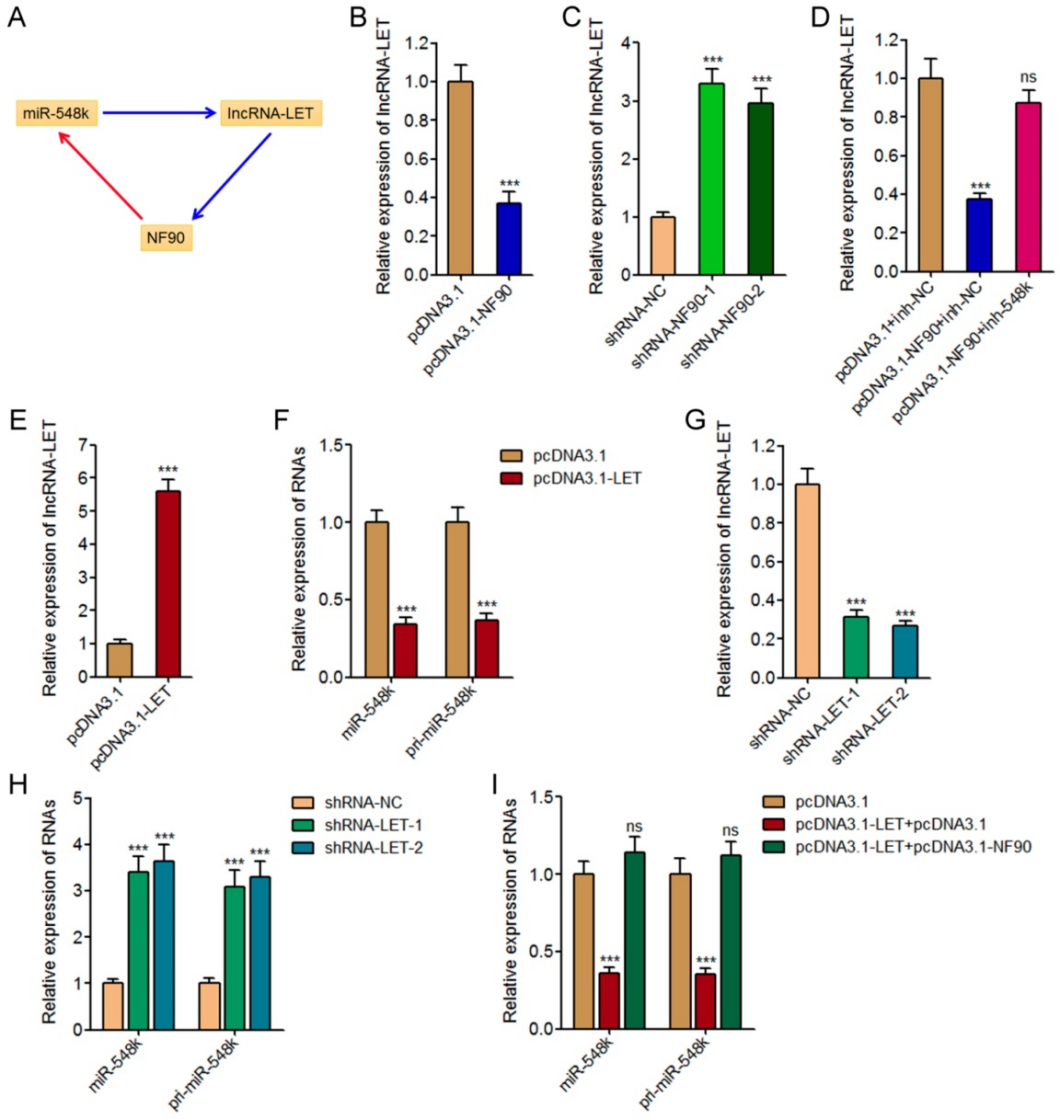
** The positive feedback loop between NF90, miR-548k, and lncRNA-LET.** (A) Schematic outline of the reciprocal regulation between NF90, miR-548k, and lncRNA-LET. Red arrowhead represents positive regulation, and while blue arrowhead represents negative regulation. (B) lncRNA-LET expression in NF90 overexpressed and control KYSE30 cells was measured by qRT-PCR. (C) lncRNA-LET expression in NF90 depleted and control Eca-109 cells was measured by qRT-PCR. (D) lncRNA-LET expression in NF90 overexpressed and miR-548k simultaneously depleted and control KYSE30 cells was measured by qRT-PCR. (E) lncRNA-LET expression in lncRNA-LET overexpressed and control Eca-109 cells was measured by qRT-PCR. (F) miR-548k and pri-miR-548k expressions in lncRNA-LET overexpressed and control Eca-109 cells were measured by qRT-PCR. (G) lncRNA-LET expression in lncRNA-LET depleted and control KYSE30 cells was measured by qRT-PCR. (H) miR-548k and pri-miR-548k expressions in lncRNA-LET depleted and control KYSE30 cells were measured by qRT-PCR. (I) miR-548k and pri-miR-548k expressions in lncRNA-LET and NF90 simultaneously overexpressed and control Eca-109 cells were measured by qRT-PCR. Results are presented as mean ± S.D. (n = 3). ***P < 0.001, ns, not significant, by Student's *t*-test (B, E and F) or one-way ANOVA followed by Dunnett's multiple comparison test (C, D, G, H and I).

In our previous study, we have found miR-548k directly targets and inhibits the expression of lncRNA-LET 21. Due to the negative regulation of NF90 by lncRNA-LET, we also found miR-548k upregulated NF90 in our previous study 39. Combined with the positive regulation of miR-548k by NF90, we supposed a feedback regulatory loop existed between NF90, miR-548k, and lncRNA-LET (**Figure 4A**).

First, we investigated the effects of NF90 on miR-548k and lncRNA-LET. The above described results have demonstrated the positive regulation of miR-548k by NF90. The effects of NF90 on lncRNA-LET were investigated in NF90 overexpressed and control KYSE30 cells, and NF90 depleted and control Eca-109 cells. As displayed in **Figure 4B** and **4C**, enhanced expression of NF90 downregulated lncRNA-LET expression, and while depletion of NF90 upregulated lncRNA-LET expression. Moreover, the expression of lncRNA-LET was evaluated in NF90 overexpressed and miR-548k simultaneously inhibited KYSE30 cells. As displayed in **Figure 4D**, inhibition of miR-548k abolished the downregulation of lncRNA-LET caused by enhanced expression of NF90. These data suggested that NF90 negatively regulated lncRNA-LET via positive regulation of miR-548k.

Second, our previous report has showed that miR-548k positively regulated NF90 via negative regulation of lncRNA-LET 21.

Third, we investigated the effects of lncRNA-LET on NF90 and miR-548k. Our previous report has revealed the negative regulation of NF90 by lncRNA-LET 21. In this study, we further investigated the effects of lncRNA-LET on miR-548k. We overexpressed lncRNA-LET in Eca-109 cells via transfecting lncRNA-LET overexpression plasmids (**Figure 4E**). Then, the expression of miR-548k and pri-miR-548k in lncRNA-LET overexpressed and control Eca-109 cells were detected. As displayed in **Figure 4F**, enhanced expression of lncRNA-LET downregulated the expression of miR-548k and pri-miR-548k. Moreover, we depleted lncRNA-LET in KYSE30 cells via transfecting lncRNA-LET specific shRNAs (**Figure 4G**). Then, the expression of miR-548k and pri-miR-548k were detected, and the results displayed that depletion of lncRNA-LET upregulated the expression of miR-548k and pri-miR-548k (**Figure 4H**). Overexpression of NF90 reversed the downregulation of miR-548k and pri-miR-548k caused by enhanced expression of lncRNA-LET (**Figure 4I**). These data suggested that lncRNA-LET negatively regulated miR-548k via downregulation of NF90. Collectively, these results demonstrated the positive feedback loop between NF90, miR-548k, and lncRNA-LET.

### The NF90/miR-548k/lncRNA-LET feedback loop controls the expression of HIF-1α, VEGF, KLF10, EGFR, and p53

**Fig 5:**
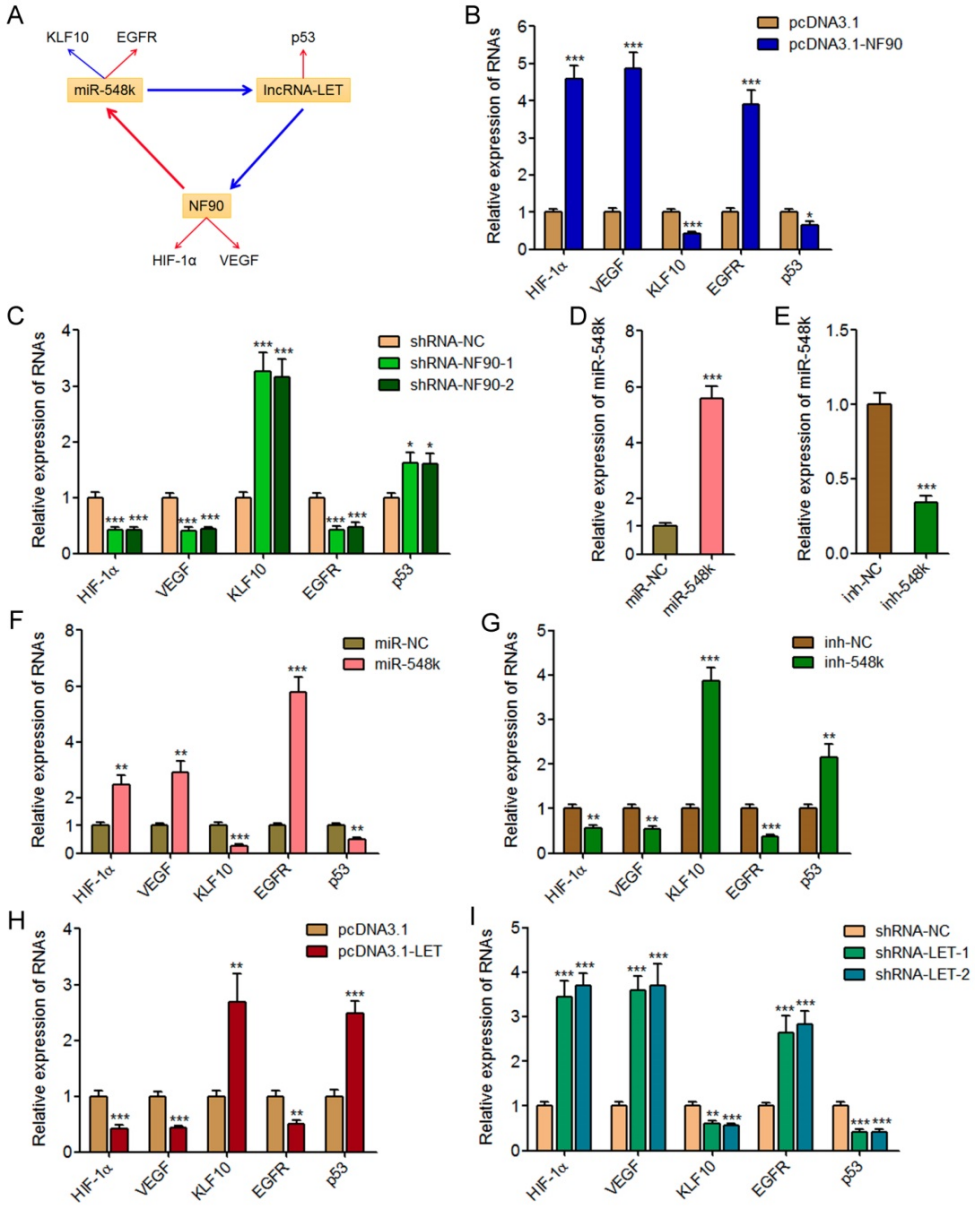
** The NF90/miR-548k/lncRNA-LET feedback loop regulates the expression of HIF-1α, VEGF, KLF10, EGFR, and p53.** (A) Schematic outline of the regulation of HIF-1α, VEGF, KLF10, EGFR, and p53 by NF90, miR-548k, and lncRNA-LET. Red arrowhead represents positive regulation, and while blue arrowhead represents negative regulation. (B) HIF-1α, VEGF, KLF10, EGFR, and p53 expressions in NF90 overexpressed and control KYSE30 cells were measured by qRT-PCR. (C) HIF-1α, VEGF, KLF10, EGFR, and p53 expressions in NF90 depleted and control Eca-109 cells were measured by qRT-PCR. (D) miR-548k expression in miR-548k overexpressed and control KYSE30 cells was measured by qRT-PCR. (E) miR-548k expression in miR-548k depleted and control Eca-109 cells was measured by qRT-PCR. (F) HIF-1α, VEGF, KLF10, EGFR, and p53 expressions in miR-548k overexpressed and control KYSE30 cells were measured by qRT-PCR. (G) HIF-1α, VEGF, KLF10, EGFR, and p53 expressions in miR-548k depleted and control Eca-109 cells were measured by qRT-PCR. (H) HIF-1α, VEGF, KLF10, EGFR, and p53 expressions in lncRNA-LET overexpressed and control Eca-109 cells were measured by qRT-PCR. (I) HIF-1α, VEGF, KLF10, EGFR, and p53 expressions in lncRNA-LET depleted and control KYSE30 cells were measured by qRT-PCR. Results are presented as mean ± S.D. (n = 3). *P < 0.05, **P < 0.01, ***P < 0.001, by Student's *t*-test (B, D, E, F, G and H) or one-way ANOVA followed by Dunnett's multiple comparison test (C, I).

NF90 has been reported to regulate hypoxia inducible factor-1α (HIF-1α) and vascular endothelial growth factor (VEGF) mRNA stability and upregulate the expression of HIF-1α and VEGF in human cancers 39, 43. miR-548k has been reported to inhibit Kruppel like factor 10 (KLF10) and activate epidermal growth factor receptor (EGFR) in ESCC 44. lncRNA-LET has been reported to upregulate p53 in ESCC 45. HIF-1α, VEGF, KLF10, EGFR, and p53 are crucial factors implicated in tumorigenesis and progression. HIF-1α, VEGF, and EGFR has oncogenic roles, and while KLF10 and p53 has tumor suppressive roles. Thus, we next investigated the effects of the NF90/miR-548k/lncRNA-LET feedback loop on HIF-1α, VEGF, KLF10, EGFR, and p53 (**Figure 5A**).

First, we measured the expression of HIF-1α, VEGF, KLF10, EGFR, and p53 in NF90 overexpressed and control KYSE30 cells, and NF90 depleted and control Eca-109 cells. As displayed in **Figure 5B** and **5C**, enhanced expression of NF90 upregulated HIF-1α and VEGF, downregulated KLF10, upregulated EGFR, and downregulated p53, which were consistent with the effects of miR-548k overexpression on KLF10 and EGFR, and also the effects of lncRNA-LET depletion on p53. Conversely, depletion of NF90 downregulated HIF-1α and VEGF, upregulated KLF10, downregulated EGFR, and upregulated p53, which were consistent with the effects of miR-548k depletion on KLF10 and EGFR, and also the effects of lncRNA-LET overexpression on p53.

Second, we constructed miR-548k overexpressed KYSE30 cells via infecting miR-548k overexpression lentiviruses, and also miR-548k depleted Eca-109 cells via infecting miR-548k inhibition lentiviruses (**Figure 5D** and **5E**). Then, we measured the expression of HIF-1α, VEGF, KLF10, EGFR, and p53 in miR-548k overexpressed KYSE30 cells and miR-548k depleted Eca-109 cells. As displayed in **Figure 5F** and **5G**, enhanced expression of miR-548k upregulated HIF-1α and VEGF, downregulated KLF10, upregulated EGFR, and downregulated p53, which were consistent with the effects of NF90 overexpression on HIF-1α and VEGF, and also the effects of lncRNA-LET depletion on p53. Conversely, depletion of miR-548k downregulated HIF-1α and VEGF, upregulated KLF10, downregulated EGFR, and upregulated p53, which were consistent with the effects of NF90 depletion on HIF-1α and VEGF, and also the effects of lncRNA-LET overexpression on p53.

Third, we measured the expression of HIF-1α, VEGF, KLF10, EGFR, and p53 in lncRNA-LET overexpressed Eca-109 cells and lncRNA-LET depleted KYSE30 cells. As displayed in **Figure 5H** and **5I**, enhanced expression of lncRNA-LET downregulated HIF-1α and VEGF, upregulated KLF10, downregulated EGFR, and upregulated p53, which were consistent with the effects of NF90 depletion on HIF-1α and VEGF, and also the effects of miR-548k depletion on KLF10 and EGFR. Conversely, depletion of lncRNA-LET upregulated HIF-1α and VEGF, downregulated KLF10, upregulated EGFR, and downregulated p53, which were consistent with the effects of NF90 overexpression on HIF-1α and VEGF, and also the effects of miR-548k overexpression on KLF10 and EGFR. Collectively, these data suggested that the NF90/miR-548k/lncRNA-LET feedback loop upregulates HIF-1α, VEGF, and EGFR, and while downregulates KLF10 and p53.

### Activation of the NF90/miR-548k/lncRNA-LET feedback loop significantly promotes ESCC cell proliferation and migration in vitro and ESCC xenograft growth in vivo

**Fig 6:**
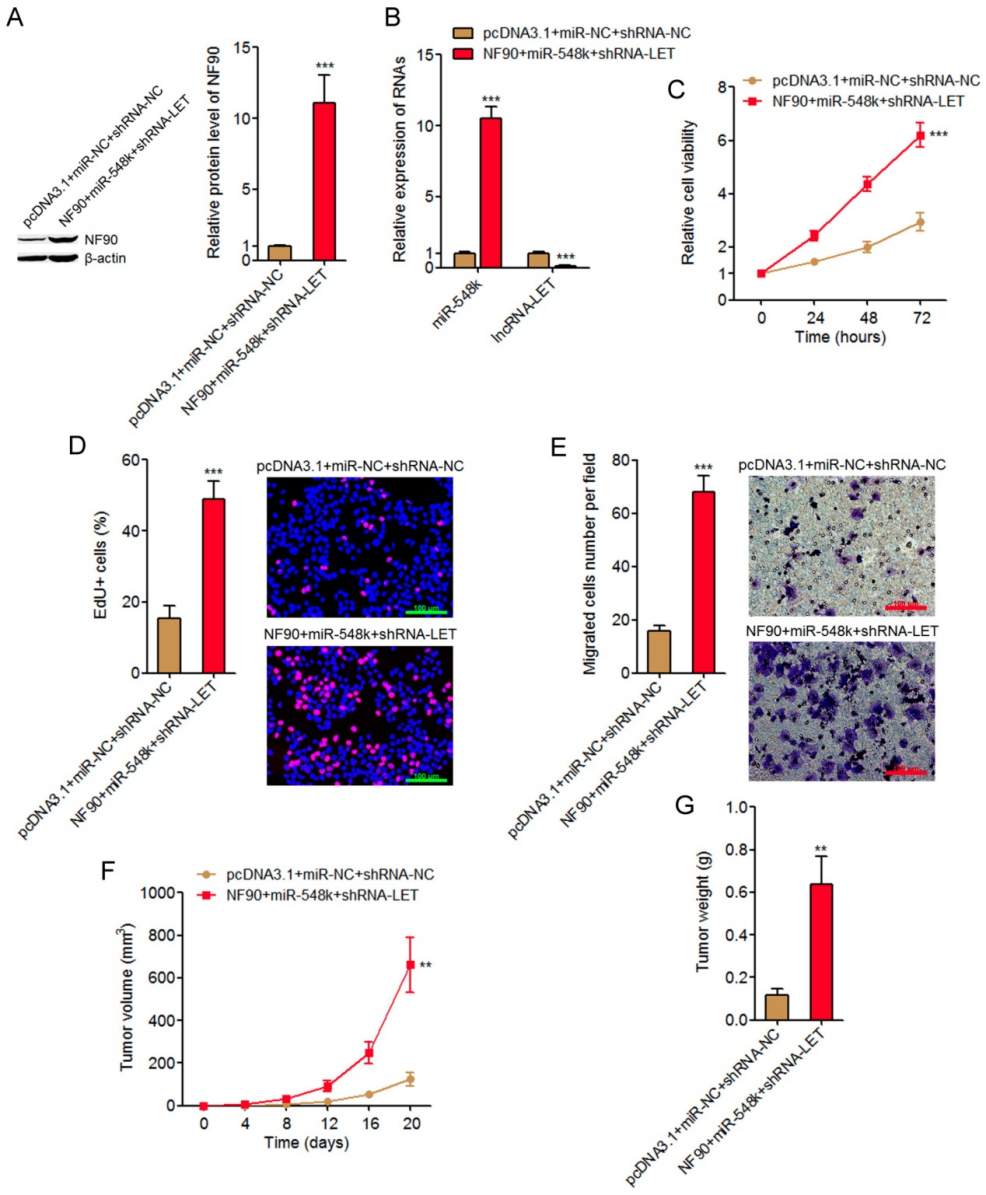
** Activation of the NF90/miR-548k/lncRNA-LET feedback loop significantly promotes ESCC progression.** (A) NF90 expressions in NF90 and miR-548k simultaneously overexpressed and lncRNA-LET simultaneously depleted KYSE30 cells were measured by western blot. (B) miR-548k and lncRNA-LET expressions in NF90 and miR-548k simultaneously overexpressed and lncRNA-LET simultaneously depleted KYSE30 cells were measured by qRT-PCR. (C) Glo cell viability assay was performed to detect cell viability of NF90 and miR-548k simultaneously overexpressed and lncRNA-LET simultaneously depleted KYSE30 cells. (D) EdU incorporation assay was performed to detect cell proliferation of NF90 and miR-548k simultaneously overexpressed and lncRNA-LET simultaneously depleted KYSE30 cells. The red color represents EdU-positive and proliferation active cells. Scale bars, 100 μm. (E) Transwell assay was performed to detect cell migration of NF90 and miR-548k simultaneously overexpressed and lncRNA-LET simultaneously depleted KYSE30 cells. Scale bars, 100 μm. For A-E, results are presented as mean ± S.D. (n = 3). ***P < 0.001 by Student's *t*-test. (F) NF90 and miR-548k simultaneously overexpressed and lncRNA-LET simultaneously depleted KYSE30 cells were subcutaneously injected into nude mice. Xenograft tumor volumes were detected every four days. (G) Xenograft tumor weights were detected at the 20^th^ days after injection. For F-G, results are presented as mean ± S.D. (n = 5 mice). **P < 0.01 by Mann-Whitney *U* test.

The NF90/miR-548k/lncRNA-LET feedback loop upregulates HIF-1α, VEGF, and EGFR, which all have oncogenic roles. The NF90/miR-548k/lncRNA-LET feedback loop also downregulates KLF10 and p53, which both have tumor suppressive roles. Therefore, we further investigated the biological roles of the NF90/miR-548k/lncRNA-LET feedback loop in ESCC. We activated the feedback loop via constructing NF90 and miR-548k simultaneously overexpressed and lncRNA-LET simultaneously depleted KYSE30 cells (**Figure 6A** and **6B**). Glo cell viability assay displayed that activation of the NF90/miR-548k/lncRNA-LET feedback loop markedly increased cell viability of KYSE30 cells (**Figure 6C**). EdU incorporation assay further displayed that activation of the NF90/miR-548k/lncRNA-LET feedback loop markedly promoted cell proliferation of KYSE30 cells (**Figure 6D**). Transwell migration assay displayed that activation of the NF90/miR-548k/lncRNA-LET feedback loop markedly promoted cell migration of KYSE30 cells (**Figure 6E**). To further explore the biological roles of the NF90/miR-548k/lncRNA-LET feedback loop in ESCC, NF90 and miR-548k simultaneously overexpressed and lncRNA-LET simultaneously depleted and control KYSE30 cells were subcutaneously injected into nude mice. Xenograft tumor growth was detected via measuring tumor volumes every four days and tumor weights at the 20^th^ day after injection. As displayed in **Figure 6F** and **6G**, activation of the NF90/miR-548k/lncRNA-LET feedback loop markedly promoted xenograft tumor growth *in vivo*. Collectively, these data supported the strongly oncogenic roles of the NF90/miR-548k/lncRNA-LET feedback loop in ESCC.

### Targeting the NF90/miR-548k/lncRNA-LET feedback loop significantly represses ESCC cell proliferation and migration in vitro and ESCC xenograft growth in vivo

**Fig 7:**
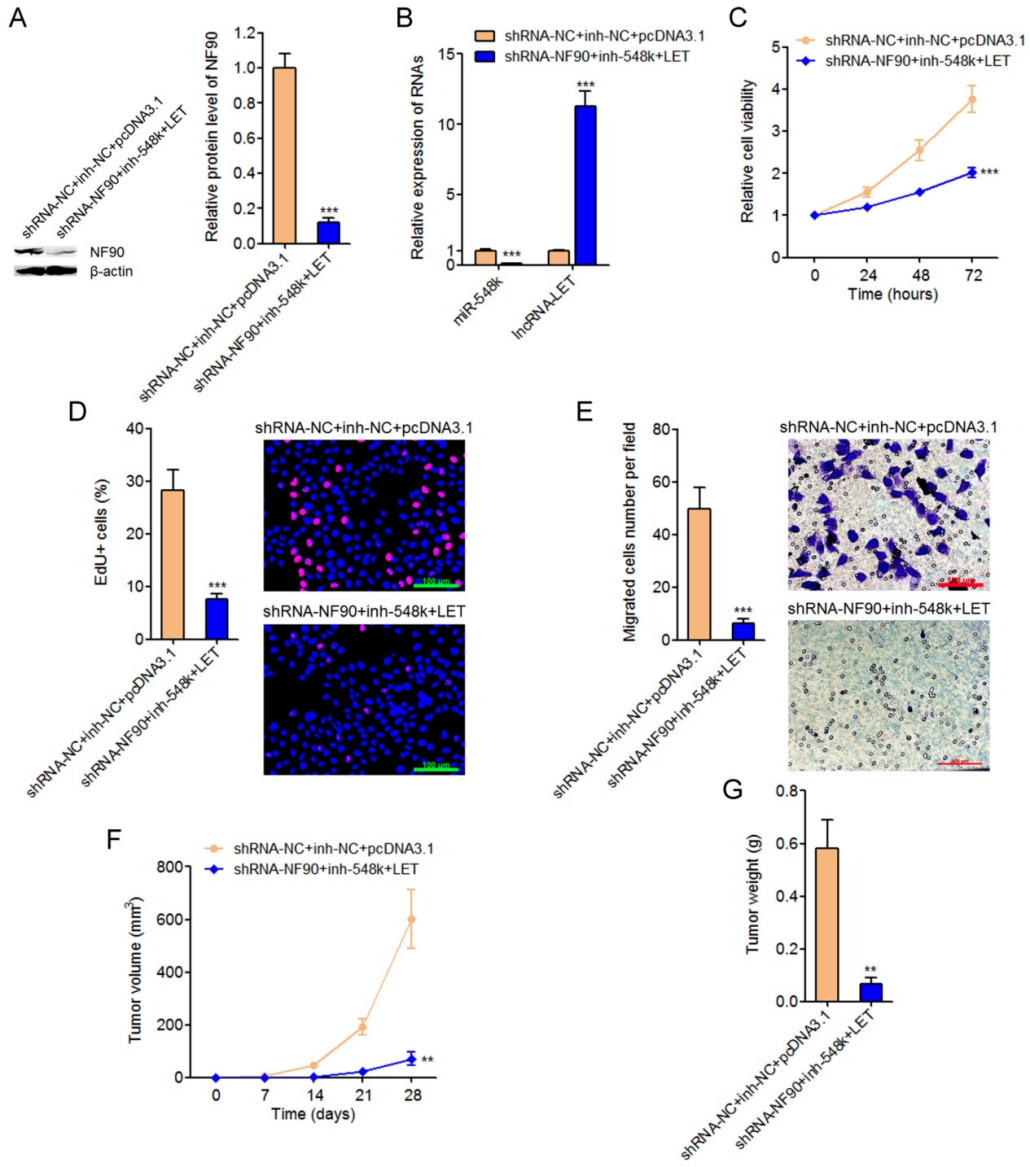
** Targeting the NF90/miR-548k/lncRNA-LET feedback loop significantly represses ESCC progression.** (A) NF90 expressions in NF90 and miR-548k simultaneously depleted, and lncRNA-LET simultaneously overexpressed Eca-109 cells were measured by western blot. (B) miR-548k and lncRNA-LET expressions in NF90 and miR-548k simultaneously depleted, and lncRNA-LET simultaneously overexpressed Eca-109 cells were measured by qRT-PCR. (C) Glo cell viability assay was performed to detect cell viability of NF90 and miR-548k simultaneously depleted and lncRNA-LET simultaneously overexpressed Eca-109 cells. (D) EdU incorporation assay was performed to detect cell proliferation of NF90 and miR-548k simultaneously depleted and lncRNA-LET simultaneously overexpressed Eca-109 cells. The red color represents EdU-positive and proliferation active cells. Scale bars, 100 μm. (E) Transwell assay was performed to detect cell migration of NF90 and miR-548k simultaneously depleted and lncRNA-LET simultaneously overexpressed Eca-109 cells. Scale bars, 100 μm. For A-E, results are presented as mean ± S.D. (n = 3). ***P < 0.001 by Student's *t*-test. (F) NF90 and miR-548k simultaneously depleted and lncRNA-LET simultaneously overexpressed Eca-109 cells were subcutaneously injected into nude mice. Xenograft tumor volumes were detected every seven days. (G) Xenograft tumor weights were detected at the 28^th^ days after injection. For F-G, results are presented as mean ± S.D. (n = 5 mice). **P < 0.01 by Mann-Whitney *U* test.

To explore the significances of targeting the NF90/miR-548k/lncRNA-LET feedback loop for ESCC, we constructed NF90 and miR-548k simultaneously depleted and lncRNA-LET simultaneously overexpressed Eca-109 cells (**Figure 7A** and** 7B**). Glo cell viability assay displayed that targeting the NF90/miR-548k/lncRNA-LET feedback loop markedly decreased cell viability of Eca-109 cells (**Figure 7C**). EdU incorporation assay further displayed that targeting the NF90/miR-548k/lncRNA-LET feedback loop markedly inhibited cell proliferation of Eca-109 cells (**Figure 7D**). Transwell migration assay displayed that targeting the NF90/miR-548k/lncRNA-LET feedback loop markedly inhibited cell migration of Eca-109 cells (**Figure 7E**). NF90 and miR-548k simultaneously depleted and lncRNA-LET simultaneously overexpressed and control Eca-109 cells were subcutaneously injected into nude mice. Xenograft tumor growth was detected via measuring tumor volumes every seven days and tumor weights at the 28^th^ day after injection. As displayed in **Figure 7F** and **7G**, targeting the NF90/miR-548k/lncRNA-LET feedback loop markedly repressed xenograft tumor growth *in vivo*. Collectively, these data demonstrated that targeted inhibition of the NF90/miR-548k/lncRNA-LET feedback loop has significantly tumor suppressive roles in ESCC.

## Discussion

The underlying molecular mechanisms mediating tumorigenesis and progression of ESCC are not fully understood 46, 47. Although many genome and transcriptome sequencings have identified many mutated genes and aberrant expressed transcripts in ESCC, the roles and regulatory mechanisms of most of these genes and transcripts in ESCC are still unclear 7, 48, 49. Some of these genes and transcripts may be driver molecular events of ESCC. But, many others may also be passenger molecular events in ESCC. Therefore, the identification of functional molecular events is critical for providing efficient therapeutic targets for ESCC.

In this study, we identified a positive feedback amplification loop between NF90, miR-548k, and lncRNA-LET. Functional assays demonstrated that activation of the NF90/miR-548k/lncRNA-LET feedback loop significantly promoted ESCC cell proliferation and migration *in vitro*, and ESCC tumor growth *in vivo*. Targeting the NF90/miR-548k/lncRNA-LET feedback loop significantly inhibited ESCC cell proliferation and migration *in vitro*, and ESCC tumor growth *in vivo*. Thus, our study provided a novel strategy to inhibit ESCC, which is the combined manipulation of a protein (NF90), a miRNA (miR-548k), and a lncRNA (lncRNA-LET).

NF90 is an RNA binding protein, which has complex and diverse biological roles and mechanisms of action in different diseases. Zhuang et al. reported NF90 promoted gemcitabine resistance of bladder cancer via repressing miR-145 biogenesis 41. Zhang et al. reported NF90 promoted angiogenesis of cervical cancer via regulating PI3K/Akt signaling pathway 39. Barbier et al. reported NF90 reduced ovarian cancer proliferation and metastasis via regulating DICER expression 50. Jiang et al. reported NF90 promoted metastasis of breast tumors via repressing p21 51. Zhou et al. reported NF90 promoted colorectal cancer progression via regulating VEGF 43. However, the roles and mechanisms of action of NF90 in ESCC are still unknown. In this study, using gain-of-function and loss-of-function assays, we found NF90 promoted ESCC cell proliferation and migration. Mechanistic investigation revealed that NF90 directly bound pri-miR-548k, increased the stability of pri-miR-548k, and upregulated the expression of pri-miR-548k and miR-548k. The positive regulation of miR-548k by NF90 is different from the reported effects of NF90 on suppressing biogenesis of miR-7 and miR-145 40, 41. The different binding manner between NF90 and different pri-miRNAs may have different effects on the processing and/or degradation of different pri-miRNAs, which need further investigation.

In our previous study, we have found the negative regulation of lncRNA-LET by miR-548k, the negative regulation of NF90 by lncRNA-LET, and thus the positive regulation of NF90 by miR-548k via repressing lncRNA-LET 21. Combination with the above identified positive regulation of miR-548k by NF90, NF90, miR-548k, and lncRNA-LET forms feedback amplification loop in ESCC. Indeed, we verified that NF90 upregulated miR-548k and downregulated lncRNA-LET, miR-548k downregulated lncRNA-LET and upregulated NF90, lncRNA-LET downregulated NF90 and miR-548k, which support the feedback loop between NF90, miR-548k, and lncRNA-LET. HIF-1α and VEGF are reported downstream targets of NF90 39. KLF10 and EGFR are reported downstream targets of miR-548k 44. p53 is reported downstream target of lncRNA-LET 45. Our results found that NF90, miR-548k, and lncRNA-LET all regulated HIF-1α, VEGF, KLF10, EGFR, and p53, which have important roles in tumor growth and metastasis. These results further support the feedback amplification loop between NF90, miR-548k, and lncRNA-LET. Due to the extensive and strong regulatory roles of the NF90/miR-548k/lncRNA-LET feedback loop in critical oncogenes and tumor suppressors, the NF90/miR-548k/lncRNA-LET feedback loop has strongly oncogenic roles in ESCC.

In conclusion, we identified a critical feedback amplification loop between NF90, miR-548k, and lncRNA-LET, which significantly promotes ESCC progression. Targeting the NF90/miR-548k/lncRNA-LET feedback loop significantly represses ESCC progression. Thus, our study suggests that the NF90/miR-548k/lncRNA-LET feedback loop may be promising therapeutic targets for ESCC.

## Abbreviations

ESCC: esophageal squamous cell carcinoma
lncRNA: long noncoding RNA
NF90: nuclear factor 90
miRNA: microRNA
cDNA: complementary DNA
qRT-PCR: quantitative real time polymerase chain reaction
RIP: RNA Immunoprecipitation
EdU: Ethynyl deoxyuridine
HIF-1α: hypoxia inducible factor-1α
VEGF: vascular endothelial growth factor
KLF10: Kruppel like factor 10
EGFR: epidermal growth factor receptor
